# Analysis of Environmental Factors Affecting the Atmospheric Corrosion Rate of Low-Alloy Steel Using Random Forest-Based Models

**DOI:** 10.3390/ma13153266

**Published:** 2020-07-23

**Authors:** Luchun Yan, Yupeng Diao, Kewei Gao

**Affiliations:** 1 School of Materials Science and Engineering, University of Science and Technology Beijing, Beijing 100083, China; lcyan@ustb.edu.cn (L.Y.); diaoyupeng@126.com (Y.D.); 2 Beijing Advanced Innovation Center for Materials Genome Engineering, University of Science and Technology Beijing, Beijing 100083, China

**Keywords:** atmospheric corrosion, low-alloy steel, atmospheric exposure test, feature importance, random forest, machine learning, data mining

## Abstract

As one of the factors (e.g., material properties, surface quality, etc.) influencing the corrosion processes, researchers have always been exploring the role of environmental factors to understand the mechanism of atmospheric corrosion. This study proposes a random forest algorithm-based modeling method that successfully maps both the steel’s chemical composition and environmental factors to the corrosion rate of low-alloy steel under the corresponding environmental conditions. Using the random forest models based on the corrosion data of three different atmospheric environments, the environmental factors were proved to have different importance sequence in determining the environmental corrosivity of open and sheltered exposure test conditions. For each exposure test site, the importance of environmental features to the corrosion rate is also ranked and analyzed. Additionally, the feasibility of the random forest model to predict the corrosion rate of steel samples in the new environment is also demonstrated. The volume and representativeness of the corrosion data in the training data are considered to be the critical factors in determining its prediction performance. The above results prove that machine learning provides a useful tool for the analysis of atmospheric corrosion mechanisms and the evaluation of corrosion resistance.

## 1. Introduction

Due to the comprehensive advantages of mechanical properties and economic cost, low-alloy steel has been widely used in various infrastructures and engineering projects. Some low-alloy steels containing Cu, Cr and Ni elements (i.e., weathering steel) showed good corrosion resistance in rural, urban, industrial, and marine atmospheric environments, and they are considered to have important application values [1,2]. As the corrosion resistance directly affects the service life and safety of related equipment and constructions, understanding the correlation between the corrosion rate and the environmental parameters is of great significance for the application and development of low-alloy steel [3,4].

At present, researchers have proved the influence of many elemental and environmental factors on metal’s corrosion behavior; many atmospheric corrosion mechanisms have been established [5,6]. Temperature and relative humidity provide conditions for the formation of a wet electrochemical corrosion environment on the surface of metals [7]. The relatively higher levels of atmospheric pollutants such as sulfur dioxide in industrial/urban environments and chlorides in marine environments distinctly accelerate the corrosion process [8,9]. However, even in the same type of atmospheric environment, the environmental corrosivity still differs because of the different intensities of environmental factors, the development of pollution and current climate conditions, etc. [10]. For instance, Ma et al. tested the stability of 09CuPCrNi steel exposed at two test sites in tropical marine environments. Although both test sites belong to the same type of atmospheric environment, significant corrosion rate differences of the steel samples was observed because of the different chloride ion deposition levels [11]. Until now, the most reliable method is still to place the materials at the target location for atmospheric exposure tests. But traditional exposure test methods are costly and time-consuming [12]. Therefore, based on limited experiments, fitting the corrosion rate and related influencing factors and establishing a corrosion rate prediction model is one of the effective ways to explore atmospheric corrosion.

Due to the development of many international organization and national cooperative projects, a large number of atmospheric corrosion tests have been carried out worldwide [13,14]. Based on accumulated corrosion data, methods like the linear model, power model, power-linear model, and bi-linear model are usually used to fit the correlation between environmental factors and corrosion rate and to analyze the importance of these factors [15,16,17,18]. Researchers have also been looking for more efficient and accurate tools for data analysis and utilization. For instance, Panchenko et al. developed a new dose-response function which provided more accurate prediction results of the first-year corrosion rate than other standard and unified dose-response functions. Quantitative estimations of the effects of each atmosphere corrosivity parameter on corrosion were considered important to develop more accurate dose-response functions [19]. Through the regression analysis of corrosion data, the in-depth exploration of atmospheric corrosion processes is accelerated. Morcillo et al. summarized the atmospheric corrosion mechanism of weathering steel, the formation of protective rust layers, and the role of alloying elements, and they made suggestions about the scientific design of new weathering steel for application in marine atmospheres [20]. Due to limitations in the regression accuracy, it is usually difficult to provide an accurate assessment for the corrosion behavior of metals in specific environments [21].

With substantial analysis and simulation capacities to multi-feature fitting problems and small datasets, machine learning has been widely used in material research [22,23]. In the field of corrosion research, machine learning has also been used for corrosion rate prediction, electrochemical test simulation, corrosion inhibitor design [24,25], etc. Wen et al. proposed a support vector regression model for prediction of the corrosion rate of 3C steel under five different seawater environmental factors, including temperature, dissolved oxygen, salinity, pH value, and oxidation-reduction potential [26]. Nevertheless, the machine learning methods are considered to be black boxes. Therefore, researchers mainly focus on modeling and prediction of the target properties [27]. For instance, Jian et al. used the artificial neural network method to distinguish the type of corrosion from electrochemical noise signals [28]. Many machine learning algorithms also have significant advantages in analyzing correlations between target variables [29,30]. For instance, Gong et al. used machine learning methods to construct polarization curves and electrochemical impedance spectra and found that the sulfide concentration had the most significant influence on the polarization curve, followed by the chloride ion concentration; the temperature influence was the smallest [31]. Therefore, machine learning is considered to play a greater role in the analysis of the influencing factors on atmospheric corrosion.

In this study, the corrosion rate of low-alloy steel under open and sheltered environments at three different atmospheric exposure test sites was estimated. The random forest algorithm was used to establish the corrosion rate prediction models, which can map the elemental and environmental factors to the corrosion rate of low-alloy steel samples. Meanwhile, the determinant factors of environmental corrosivity and the importance of the environmental factors in each exposure test site were analyzed. In order to investigate the feasibility of applying the corrosion rate prediction model in new environments, the factors affecting the prediction accuracy of the random forest model were demonstrated and discussed. Based on the above results, this study aims to prove the advantages of machine learning methods in corrosion research and provide effective methods for corrosion data utilization.

## 2. Materials and Methods 

### 2.1. Corrosion Data and Exposure Test Environments

Corrosion data of thirteen different low-alloy steel were collected from the National Institute of Materials Science (NIMS) Materials Database (MatNavi) [32]. In the original dataset, the content of some trace alloy elements was not recorded (or recorded below a detection threshold). Here, we used the minimum detection threshold of an element in the dataset as the detection content to replace the above-mentioned records. The chemical compositions of low-alloy steel are listed in Table 1.

The specimens were tested at three different atmospheric exposure test sites, which are representative geographic locations in Japan: Tsukuba (rural environment), Choshi (rural/coastal environment), and Miyakojima (subtropical coastal environment). At each exposure test site, one open and one sheltered test environment was arranged. Under the open environment, the specimens were set up horizontally as well as on a surface with 45-degree inclination to the south. Under the sheltered environment, the specimens were horizontally set up under the roof of the shelter. In addition, the open and sheltered environments at each exposure test site had precisely the same environmental conditions. The exposure tests, including test specimen preparation, requirements for exposure test sites, exposure test apparatus and measurement of environmental factors were all performed according to the standard method [33]. The exposure test was started in 1998 for all the Fe-Ni and Fe-Cr samples, in 2003 for all the Fe-Cu samples, and in 2002 for all the SM490A, SMA490 and SPA-H samples. In this study, the results of the 1-, 2-, 3- and 10-year exposure tests were collected. During the exposure test period, eight environmental parameters were monitored, and their annual averages were individually calculated. For each exposure test site, Table 2 lists the range of the annual mean environmental parameters of all the 1-, 2-, 3- and 10-year specimens.

**Figure 1:**
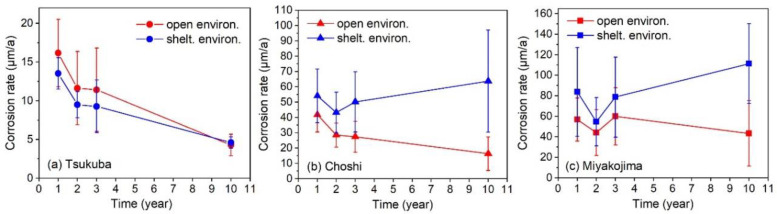
Mean corrosion rate of 13 different low-alloy steels versus exposure time. All the specimens were tested under the open and sheltered environments at the exposure test site (**a**) Tsukuba, (**b**) Choshi and (**c**) Miyakojima, individually.

The corrosion rate (annual corrosion depth calculated from the weight loss of base metal after removing the corrosion products; micrometre per year, μm/a) of steel samples versus exposure time is plotted in Figure 1. Each dot denotes the mean corrosion rate of all thirteen low-alloy steels, and the error bar equals their standard deviation. Except for a few completely corroded specimens (e.g., the 10-year specimen of Fe-1Ni and Fe-1Cr steel in Miyakojima), the corrosion data of 306 specimens (153 samples each under the open and sheltered environments) were collected.

### 2.2. Random Forest Algorithm

The random forest (RF) algorithm is a collection of decision trees to perform classification or regression analysis. Each decision tree (i.e., classification and regression trees, CART) starts from a root node and consists of multiple layers of nodes. Its input data is a subset of the training data, drawn randomly from the original dataset [34]. Every node corresponds to a feature and has a threshold. The input data are divided into two subsets according to their response to the node. Then, each subset is individually transferred to the next node (e.g., left and right child-node). The input data for a decision tree are tested and split repeatedly until they reach the terminal node, while the terminal node outputs a specific value. Since each tree is assigned a different subset of the training data and features, the RF becomes a forest of different decision trees. After training based on the training data, the hyperparameters (e.g., number of decision trees, number of features assigned to each decision tree, etc.) of the RF are optimized to mapping the input data to its target outcome. When the test data is fed to all the decision trees, the prediction results are obtained by averaging all the tree outputs (in regression tasks). Due to the existence of multiple decision trees, the robustness of the RF model is enhanced by pooling all these decision trees.

As an important by-product in the training of the RF model, the importance score provides a ranking of all features [35]. In the regression analysis, the variance reduction is used as a selection criterion; the impurity decrease strategy is employed to calculate the variances. At each node within the decision tree, the impurity (i.e., *G*(*x,v*) in Equation (1)) is calculated to measure how well a potential split is separating the samples of the two classes in this particular node.

(1) $$G\left(x,v\right)=\frac{1}{N_{s}}\left(\underset{y_{i}\in X_{left}}{\sum }{\left(y_{i}-{\bar{y}}_{left}\right)}^{2}+\underset{y_{i}\in X_{right}}{\sum }{\left(y_{i}-{\bar{y}}_{right}\right)}^{2}\right)$$

where, *x* is the feature to be split on the node; *v* is the threshold value; *N_s_* is the number of input data in the node; *X_left_* and *X_right_* are the subset data in the left and right child-nodes; *y_i_* is the target value of samples; $\bar{y}$*_left_* and $\bar{y}$*_right_* are the mean target value of samples in the left and right child-nodes. Then, the importance of a node can be calculated as:

(2) $$n_{k}=w_{k}\cdot G_{k}-w_{left}\cdot G_{left}-w_{right}\cdot G_{right}$$

where, *w_k_*, *w_left_* and *w_right_* are the ratio between sample quantities in node *k*, the left child-node, the right child-node and the total training samples quantity; *G_k_*, *G_left_*, and *G_right_* are the impurities of node *k* and the two child-nodes. For a forest, the importance of a feature can be calculated as:

(3) $$f_{i}=\frac{\sum_{j\in nodes\;split\;on\;feature\;i}n_{j}}{\sum_{k\in all\;nodes}n_{k}}$$

In order to make the sum of all features’ importances equal to 1, the importance is normalized:

(4) $$f_{i}^{\prime }=\frac{f_{i}}{\sum_{j\in all\;features}f_{j}}$$

The importance indicates how much the feature affects the prediction results. It is often used as an efficient tool in the feature screening and correlation analysis [36]. 

### 2.3. Model Setting and Performance Evaluation

In this study, a total of seventeen features were set as the input of corrosion rate prediction models: eight elemental features, C, Si, Mn, P, S, Cu, Cr, Ni (wt.%); eight environmental features (Table 2); and the exposure time (year). The corrosion rate (μm/a) was the target output. The coefficient of determination (*R^2^*) and mean absolute error (*MAE*) were used to evaluate the accuracy of the corrosion rate prediction model [37]. They are formulated as:

(5) $$R^{2}=1-\frac{SS_{res}}{SS_{tot}}=1-\frac{\sum_{i=1}^{n}{\left(y_{i}-f_{i}\right)}^{2}}{\sum_{i=1}^{n}{\left(y_{i}-\bar{y}\right)}^{2}}$$

(6) $$MAE=\frac{1}{n}\sum_{i=1}^{n}\left|f_{i}-y_{i}\right|$$

where *SS_res_* denotes the residual sum of squares; *SS_tot_* denotes the total sum of squares; *n* is the number of samples; *f_i_* is the predicted corrosion rate; *y_i_* is the target corrosion rate; and $\bar{y}$ is the mean target corrosion rate of all samples. Larger values of *R*^2^ normally indicate higher prediction accuracy. The *MAE* can better reflect the actual situation of the prediction error.

All the regression analysis work was conducted using Anaconda (an open-source Python distribution platform), TensorFlow and the scikit-learn toolkit.

## 3. Results and Discussion

### 3.1. Random Forest Prediction Model

**Figure 2:**
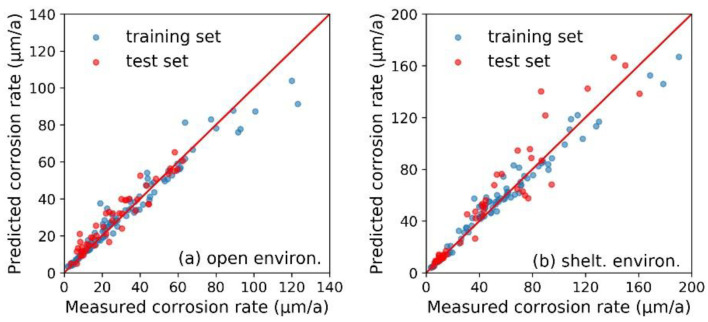
The random forest algorithm-based corrosion rate prediction models for steel samples under (**a**) open environment and (**b**) sheltered environment at the exposure test sites of Tsukuba, Choshi and Miyakojima.

As shown in Figure 2, the RF models were established on the basis of corrosion data at the exposure test sites of Tsukuba, Choshi, and Miyakojima. Due to the reported conclusions about the significant difference of steel’s corrosion behavior under open (i.e., unsheltered) and sheltered environments, we established the RF model for open and sheltered environments, respectively [38].

Based on 153 specimens under open (or sheltered) environment at the exposure test sites of Tsukuba, Choshi and Miyakojima, the corrosion data were randomly divided into a training set (70% of total, 107 samples) and a test set (30% of total, 46 samples). During training, the RF model was supposed to catch the relationship between the 17 input features (i.e., eight elemental features, listed in Table 1; eight environmental features, listed in Table 2; and the exposure time) and the corrosion rate, based on the data contained in the training set. The RF model then acquired the ability to predict the corrosion rate, and was verified by the test sets. As listed in Table 3, the *R^2^* and *MAE* results of each RF model in response to both the training set and the test set were also calculated. Basically, the RF model showed similar prediction accuracy for both training and test set samples. However for samples with a high corrosion rate under both open (>80 μm/a) and sheltered (>100 μm/a) environments, the prediction accuracy was slightly lower than other samples (Figure 2). This might be due to the imbalanced data distribution, which limited the fitting effect of the machine learning model on these data [39]. Usually, this problem can be solved by adding more related data in the training set. The above results confirmed that the random forest algorithm successfully achieved the mapping between the input features and the corrosion rate, and correctly grasped the correlation between them. Therefore, the RF model was considered to have reliable modeling capability and could support the subsequent analysis in this study.

### 3.2. Determining the Factors of Environmental Corrosivity

**Figure 3:**
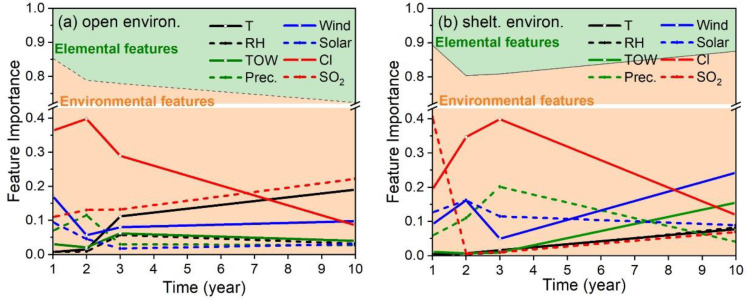
Stacked area charts of elemental and environmental feature importance, and line plots of individual environmental features. The feature importances derived from the random forest models were established on the basis of steel samples in 1-, 2-, 3- and 10-year exposure tests under the (**a**) open and (**b**) sheltered environment.

In order to explore the determinants of the environmental corrosivity among the atmospheric environments at Tsukuba, Choshi, and Miyakojima, all the corrosion data under the open environment were divided into four subsets according to the exposure time of the steel samples (i.e., 1-, 2-, 3- and 10-year). Each subset included the data from all three exposure test sites. The RF models were established for each subset of corrosion data (input features: 8 elemental and 8 environmental features; here, the exposure time became a constant and was excluded), and the corresponding feature importance results were summarized. As shown in the stacked area chart in Figure 3a, the green area denotes the sum of all eight alloying elements’ feature importance values and the orange area is the sum of all eight environmental parameters’ feature importance values. Here, the sum of elemental and environmental features’ importance values always equals to 1.0 (Equation (4)). It can be seen that the sum of all elemental feature importances increased from 0.15 to almost 0.25 in ten years of exposure. In other words, the influence of environmental factors was slightly decreased in a long-term exposure. As reported in the literature, the protective rust layer formed on the surface of low-alloy steel usually helps improve its corrosion resistance in the atmospheric environment [18]. It also could be confirmed from the results in Figure 1. In all three exposure test sites, the mean corrosion rate of thirteen different low-alloy steel gradually decreased along with exposure time. 

Meanwhile, the importance of each environmental feature was drawn in the line plot (Figure 3a). For these environmental features, their importance value changed with exposure time. This might be due to the different values of the environmental factor or the role of the rust layer on the sample surface. For convenience, we employed the mean importance value of each environmental feature to find out the key environmental factors that determine the environmental corrosivity. As depicted in Figure 3a, the Cl^−^ deposition rate, SO_2_ deposition rate, air temperature and wind speed were the four most important environmental factors that determined the environmental corrosivity. Belonging to the subtropical coastal environment, Miyakojima has the highest Cl^−^ deposition rate, air temperature, and wind speed (Table 2). Choshi, the rural/coastal environment, has the highest SO_2_ deposition rate and high Cl^−^ deposition rate and air temperature. Tsukuba (rural environment) has the mildest climate. Consistent with the level of these environmental factors (which were the determinants of environmental corrosivity), the mean corrosion rate of the steel samples also decreased sequentially from Miyakojima to Choshi to Tsukuba (Figure 1).

According to the above method, the results of steel samples under the sheltered environment are depicted in Figure 3b. Unlike the open environment, the sum of environmental parameters’ feature importances always remained above 0.8 and even slightly increased in the long-term exposure. According to the literature, surfaces sheltered from the sun and rain tend to form a loose and non-compact oxide, while surfaces openly exposed to the sun and rain produce strong adherent layers [18]. Thus, the protectiveness of the rust layer formed under the sheltered environment was low. As shown in the line plots, the four determining factors of environmental corrosivity under the sheltered environment were: Cl^−^ deposition rate, precipitation, wind speed, and solar radiation. In addition to the dominating role of chloride ions, precipitation helped keep the rust film wet and the wind accelerated the transfer of chlorides from seawater to the sample surface [40]. Since the roof blocked the deposition of particulate matter in the air (common attachments of sulfur dioxide), the actual deposition rate of sulfur dioxide on the sample surface was significantly reduced. Meanwhile, the roof protects the sample surface from rain. As a result, the dust containing sulfur dioxide can be better accumulated on the sample surface. Therefore, reasons for the changes of sulfur dioxide’s importance in long-term exposure need to be experimentally demonstrated. At the same time, reasons for the distinct difference of solar radiation’s importance rating between open and sheltered environments also need to be further demonstrated. Consistent with the corresponding level of the above four dominating environmental factors (Table 2), the environmental corrosivity at the exposure test sites from strong to weak was: Miyakojima, Choshi, Tsukuba (Figure 1). However, in Miyakojima and Choshi, the corrosion rate of steel samples gradually increased over the long term of exposure. This phenomenon might be due to the accumulation of chlorides in the loose rust layer, which was never washed away [18]. The chlorides could easily reach the substrate surface and form very corrosive environments when the rust layer was wet.

### 3.3. Determining the Factors of Corrosion Rate in Different Exposure Test Sites

Compared with the environmental differences at different exposure test sites, the environmental parameters at a specific location usually fluctuated within certain ranges. Therefore, analyzing the ranking of environmental feature importance is also helpful to understand the corrosion behavior of steel in a particular location. The thirteen types of steel were divided into three batches, and their exposure tests at each test site started in different years (i.e., each test site had three different groups of environmental parameters). We divided the corrosion data into six subsets according to the exposure test site and open/sheltered environment. For each subset of corrosion data, an RF model was established and the feature importance was summarized.

**Figure 4:**
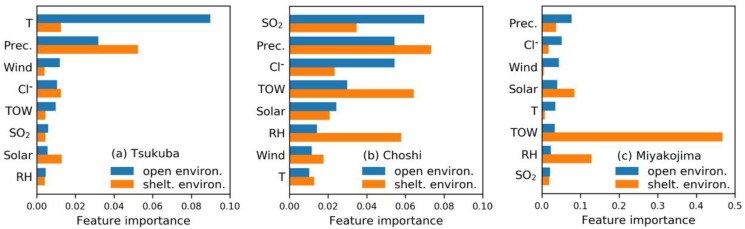
The environmental feature importance on the corrosion rate of steel samples under the open and sheltered environment at the exposure test site (**a**) Tsukuba, (**b**) Choshi, and (**c**) Miyakojima.

As shown in Figure 4, the feature importance sequence under the open environment was closely related to the climate characteristics of the exposure test site. The deposition rates of chlorides and SO_2_ were low in Tsukuba (Table 2). Then, the corrosion rate is more affected by the change of factors like air temperature and precipitation which lead to a time of wetness and provided a wet environment on the substrate surface for electrochemical corrosion. Choshi had the highest SO_2_ deposition rate and high chloride deposition rate. Miyakojima had the highest chloride deposition rate, and the wind, and solar radiation also provided the extra pathways of chlorides transfer. Meanwhile, the high levels of precipitation directly influenced the wet condition of the rust layer. So, the samples at these test sites are sensitive to the corresponding factors. 

Unlike the open environment, the environmental feature sequence of steel samples under the sheltered environment did not show a strong correlation with the climate characteristics of the test site. Although steel samples under both the open and sheltered environments were tested in the same locations, the roof of the shelter significantly changed the actual environmental parameters (e.g., precipitation, chloride deposition rate, etc.) near the steel samples. At first, the corrosion rate of the steel samples was still mainly determined by the levels of corresponding chloride ions and SO_2_ deposition rates (Figure 1). The content of accumulated chlorides in the rust layer was still directly determined by the deposition rate in the corresponding environment. Secondly, the sheltered environment made the steel samples in a specific test site became more sensitive to factors like precipitation, time of wetness, and RH. The sheltered environment might reduce the direct effect of changes in the chloride deposition rate. However, the loose rust layer became more sensitive to the changes of factors like precipitation, time of wetness, and relative humidity, which would promote condensation and storage of water inside the rust layer.

### 3.4. Generalization Ability of the Corrosion Rate Prediction Model

Compared with conventional time-consuming and costly atmospheric exposure tests, the corrosion rate prediction model was more effective. In this study, we investigated the factors that affected the generalization ability of corrosion rate prediction models. The corrosion data of Tsukuba and Miyakojima were employed in the model training, and the corrosion data of Choshi were used to verify the model’s generalization ability. Due to the significant differences in the corrosion behavior of the steel samples, we established corrosion rate prediction models for the open and sheltered environments, respectively. Besides the RF algorithm, the support vector regressor (SVR) and an artificial neural network (ANN) were also employed to compare their generalization abilities. As listed in Table 4, the overall performance of the RF model was better. 

**Figure 5:**
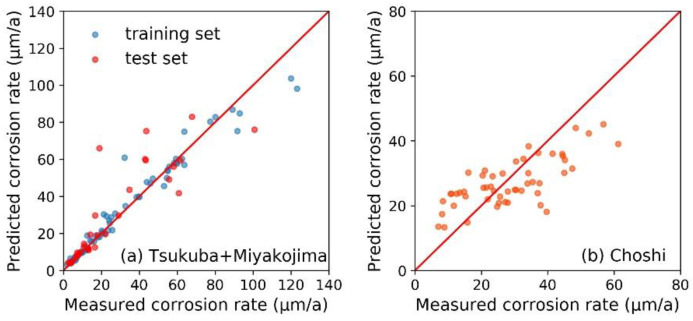
The random forest algorithm-based corrosion rate prediction model for steel samples under the open environment. (**a**) The model was trained and tested by the corrosion data of Tsukuba and Miyakojima. (**b**) The model’s generalization ability was evaluated by verifying its prediction accuracy to the same steel samples in Choshi.

The corrosion rate prediction results of the RF model under the open environment are depicted in Figure 5. For the corrosion data of Tsukuba and Miyakojima, the model showed good prediction accuracy to both the training and test sets. It means that that the RF algorithm has successfully achieved an accurate mapping between the input features (i.e., elemental and environmental factors) and the corrosion rate of steel samples. When applying the RF model to a new test site (i.e., Choshi), it still exhibited a reasonable prediction accuracy. This may be due to the fact that under the open environment, the steel samples of Choshi had similar corrosion behavior with that at Tsukuba and Miyakojima (i.e., the corrosion rate decreases with exposure time, as seen in Figure 1). The climatic conditions in Choshi are basically between those of Tsukuba and Miyakojima (Table 2). In other words, the training data (from Tsukuba and Miyakojima) were representative and supported the predictive performance of the RF model in Choshi.

**Figure 6:**
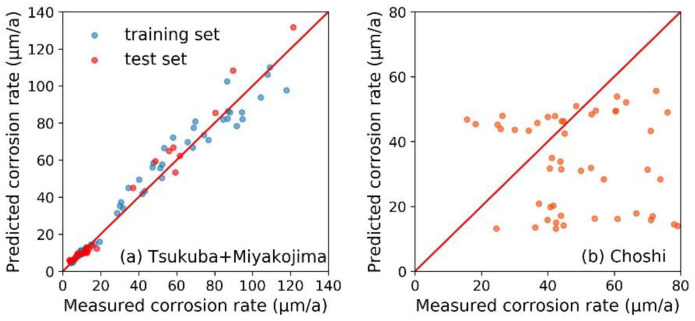
The random forest algorithm-based corrosion rate prediction model for steel samples under the sheltered environment. (**a**) The model was trained and verified by the corrosion data of Tsukuba and Miyakojima. (**b**) The model’s generalization ability was evaluated by verifying its prediction accuracy to the same steel samples in Choshi.

Figure 6 shows the results of the RF model for steel samples under the sheltered environment. Although the steel samples of Tsukuba and Miyakojima had different corrosion behavior (Figure 1), the RF model still successfully made accurate predictions to both the training and test sets. But the prediction accuracy became very poor when applying the RF model to the steel samples of Choshi. This may be due to the fact that under the sheltered environment, only the steel samples of Choshi and Miyakojima had similar corrosion behavior (Figure 2). There was also a relatively significant difference between the climate conditions of Choshi and Miyakojima. Therefore, the representativeness of the training set was found to be insufficient. Therefore, it limited the generalization ability of the RF model severely.

### 3.5. Factors Affecting the Regression Analysis Results

The machine learning algorithm showed a strong capacity in the regression analysis of multiple variables. Both the elemental and environmental factors were successfully mapped into the corrosion rate of low-alloy steel samples. Based on that, the corrosion rate of a steel sample can directly be predicted by inputting its chemical composition and the environmental parameters into the model. The models can also be used in the evaluation of environmental corrosivity and the analysis of a specific environmental factor’s influence. Especially for big datasets and statistical analysis issues in corrosion research, the machine learning methods provide a more effective method than traditional regression analysis. Due to the advantages of machine learning in multi-features regression and small data modeling, it comprises a useful tool in the use of corrosion data from various geographic locations and environments. 

As a modern data processing tool, the essence of machine learning lies in digging into useful information from the dataset. Therefore, the reliability of the analysis results is significantly dependent on the representativeness and accuracy of the raw data. In this study, we did not discuss the specific importance sequence of environmental factors (Figure 4). As the volume of the corrosion data at each exposure test site is very limited, the particular ranking of features with small importance values is sensitive to the raw data and model optimization. Thus, more relevant corrosion data must be added before an in-depth analysis can be performed. When verifying the generalization ability of the RF model, the prediction accuracy is also not satisfactory for steel samples under both the open and sheltered environments in Choshi. According to the scientific knowledge, the steel samples showed different corrosion mechanisms in the three exposure test sites which are characterized as typical rural, rural/coastal and subtropical coastal environments. The volume and representativeness of the current corrosion data were obviously insufficient to support the model for a wide range of uses. Accumulating more experimental data or comprehensively using corrosion data from multiple sources is a feasible way to enrich the dataset. However, the precision of corrosion data, which regards the measurement methods of the environmental factors and corrosion rate, should always be kept in mind when collecting and utilizing the corrosion data.

## 4. Conclusions

Based on the corrosion data of thirteen different low-alloy steels under open and sheltered environments at the exposure test sites of Tsukuba (rural environment), Choshi (rural/coastal environment) and Miyakojima (subtropical coastal environment), the random forest algorithm was used to establish the prediction of the corrosion rate. The random forest model successfully mapped 17 input features (i.e., eight elemental features, eight environmental features, and the exposure time) to the corrosion rate, and achieved accurate corrosion rate prediction. Depending on the feature importance results, the determinant factors of the environmental corrosivity were: Cl^−^ deposition rate, SO_2_ deposition rate, air temperature and wind speed for the open environment, and Cl^−^ deposition rate, precipitation, wind speed and solar radiation for the sheltered environment. At each specific exposure test site, the environmental feature importance sequence under the open environment was found to be closely related to the corresponding climate characteristics. Under the sheltered environment, the steel samples were more sensitive to factors like precipitation, time of wetness and RH. Besides the ability to analyze the corrosion behavior, the random forest model could also be used as a corrosion rate prediction tool. The volume and representativeness of the training data were supposed to be an important guarantee for the model’s generalization ability. Based on the above conclusions, the machine learning algorithms were considered to be promising tools in corrosion data utilization.

## Figures and Tables

**Table 1:** Chemical compositions (wt.%) of selected low-alloy steel from the MatNavi database.

Steel	C	Si	Mn	P	S	Cu	Cr	Ni
Fe-1Ni	0.0010	0.0030	0.0100	0.0003	0.0001	0.0090	0.0050	0.9800
Fe-3Ni	0.0010	0.0030	0.0100	0.0005	0.0002	0.0090	0.0050	3.0200
Fe-5Ni	0.0010	0.0030	0.1100	0.0006	0.0003	0.0090	0.0050	5.0100
Fe-1Cr	0.0050	0.0030	0.0700	0.0010	0.0002	0.0090	1.0100	0.0030
Fe-3Cr	0.0060	0.0030	0.0500	0.0007	0.0001	0.0090	3.0500	0.0030
Fe-5Cr	0.0030	0.0030	0.1100	0.0003	0.0010	0.0090	5.0300	0.0030
Fe-0.4Cu	0.0010	0.0030	0.0030	0.0006	0.0007	0.4300	0.0050	0.0030
Fe-1Cu	0.0011	0.0030	0.0030	0.0002	0.0001	1.0000	0.0050	0.0030
Fe-2Cu	0.0011	0.0030	0.0030	0.0002	0.0001	1.9800	0.0050	0.0030
Fe-3Cu	0.0013	0.0030	0.0030	0.0002	0.0001	2.9700	0.0050	0.0030
SM490A	0.1400	0.2500	1.3500	0.0120	0.0030	0.0090	0.0400	0.0030
SMA490	0.1300	0.2600	1.0100	0.0110	0.0050	0.3200	0.4800	0.1000
SPA-H	0.0900	0.4300	0.3800	0.1020	0.0050	0.3000	0.6700	0.1800

**Table 2:** Annual mean environmental parameters of all the 1-, 2-, 3- and 10-year specimens at the three atmospheric exposure test sites.

Environmental Parameter	Exposure Test Site			Description
	Tsukuba	Choshi	Miyakojima	
T	14.5–15.5	14.7–15.3	23.9–24.0	°C; mean air temperature
RH	74.6–77.5	77.6–79.0	78.5–79.0	%; mean relative humidity
TOW	4088–4670	4629–4908	5113–5249	h; time of wetness
Precipitation	1103–1344	1511–1791	1899–2314	mm; precipitation
Wind	1.5–2.5	3.0–3.5	4.2–4.7	m/s; mean velocity of wind
Solar	4183–6274	4193–4901	5229–5260	MJ/m^2^; solar radiation
Cl^‒^	2.8–3.3	32.0–32.3	45.8–49.2	mg NaCl/m^2^·d; chloride deposition rate
SO_2_	3.7–5.3	4.9–5.1	2.1–2.4	mg SO_2_/m^2^·d; SO_2_ deposition rate

**Table 3:** The predictive accuracy of random forest models for steel samples under open and sheltered environments at the exposure test sites of Tsukuba, Choshi, and Miyakojima.

Samples	*R^2^*		*MAE* (μm/a)	
	Training Set	Test Set	Training Set	Test Set
Open environ.	0.95	0.89	3.3	4.6
Shelt. environ.	0.97	0.86	4.3	10.0

**Table 4:** The predictive accuracy of the random forest (RF), support vector regressor (SVR), and artificial neural network (ANN) models for steel samples under open and sheltered environments. The samples in the exposure test sites of Tsukuba and Miyakojima were used to train the models and verify their prediction accuracy. The samples in the exposure test site of Choshi were used to demonstrate the generalization ability of the models.

Environment & Algorithm		*R^2^*			*MAE* (μm/a)		
		Tsukuba + Miyakojima		Choshi Evaluation Set	Tsukuba + Miyakojima		Choshi Evaluation Set
		Training Set	Test Set		Training Set	Test Set	
Open environ.	RF	0.96	0.70	0.50	3.1	7.6	8.0
	SVR	0.77	0.74	0.39	8.8	10.9	8.6
	ANN	0.81	0.68	0.31	8.4	8.7	8.9
Sheltered environ.	RF	0.97	0.92	−1.45	4.6	6.1	26.2
	SVR	0.90	0.78	0.03	12.9	17.3	17.4
	ANN	0.90	0.79	−1.77	7.7	12.0	31.8
